# PET Imaging of Fructose Metabolism in a Rodent Model of Neuroinflammation with 6-[^18^F]fluoro-6-deoxy-D-fructose

**DOI:** 10.3390/molecules27238529

**Published:** 2022-12-03

**Authors:** Amanda J. Boyle, Emily Murrell, Junchao Tong, Christin Schifani, Andrea Narvaez, Melinda Wuest, Frederick West, Frank Wuest, Neil Vasdev

**Affiliations:** 1 Azrieli Centre for Neuro-Radiochemistry, Brain Health Imaging Centre, Centre for Addiction and Mental Health, 250 College St., Toronto, ON M5T 1R8, Canada; 2 Department of Psychiatry, University of Toronto, 250 College St., Toronto, ON M5T 1R8, Canada; 3 Department of Chemistry, University of Alberta, Edmonton, AB T6G 2N4, Canada; 4 Department of Oncology, University of Alberta, Edmonton, AB T6G 1Z2, Canada

**Keywords:** fructose, neuroinflammation, PET, fluorine-18, GLUT5, microglia, FDG, FDF

## Abstract

Fluorine-18 labeled 6-fluoro-6-deoxy-D-fructose (6-[^18^F]FDF) targets the fructose-preferred facilitative hexose transporter GLUT5, which is expressed predominantly in brain microglia and activated in response to inflammatory stimuli. We hypothesize that 6-[^18^F]FDF will specifically image microglia following neuroinflammatory insult. 6-[^18^F]FDF and, for comparison, [^18^F]FDG were evaluated in unilateral intra-striatal lipopolysaccharide (LPS)-injected male and female rats (50 µg/animal) by longitudinal dynamic PET imaging in vivo. In LPS-injected rats, increased accumulation of 6-[^18^F]FDF was observed at 48 h post-LPS injection, with plateaued uptake (60–120 min) that was significantly higher in the ipsilateral vs. contralateral striatum (0.985 ± 0.047 and 0.819 ± 0.033 SUV, respectively; *p* = 0.002, n = 4M/3F). The ipsilateral–contralateral difference in striatal 6-[^18^F]FDF uptake expressed as binding potential (*BP*_SRTM_) peaked at 48 h (0.19 ± 0.11) and was significantly decreased at one and two weeks. In contrast, increased [^18^F]FDG uptake in the ipsilateral striatum was highest at one week post-LPS injection (*BP*_SRTM_ = 0.25 ± 0.06, n = 4M). Iba-1 and GFAP immunohistochemistry confirmed LPS-induced activation of microglia and astrocytes, respectively, in ipsilateral striatum. This proof-of-concept study revealed an early response of 6-[^18^F]FDF to neuroinflammatory stimuli in rat brain. 6-[^18^F]FDF represents a potential PET radiotracer for imaging microglial GLUT5 density in brain with applications in neuroinflammatory and neurodegenerative diseases.

## 1. Introduction

Neuroinflammation occurs in response to viral or bacterial infections, toxins, as well as injury to the central nervous system and involves the activation of innate immune glial cells. Prolonged neuroinflammation is a common feature linked to neurodegenerative diseases such as Alzheimer’s disease (AD) that has been corroborated by molecular imaging studies using positron emission tomography (PET) [1,2,3]. The most common PET imaging biomarker of neuroinflammation is the 18 kDa translocator protein (TSPO) [4], which is not specifically expressed on microglia, but is also found on astrocytes [5,6,7]. Nonetheless, PET imaging studies targeting TSPO, both in animal models and in humans, have shown neuroinflammation to be an early event in AD pathogenesis [8,9]. A novel PET radiotracer capable of specifically imaging microglia would be critical to further our mechanistic understanding of the link between neuroinflammation and neurodegenerative diseases in the living human brain [10,11,12,13,14].

More specific molecular targets for microglial imaging are highly sought after. Macrophage colony stimulating factor-1 receptor (CSF-1R) is one such target, which is predominantly found on microglia in the brain, with low-level expression occurring in neurons [15,16]. Several efforts to develop potent and selective CSF-1R targeted PET radiotracers for neuroimaging in preclinical and human studies are underway [17,18,19,20,21,22,23,24,25]. Two other targets of particular interest for PET imaging microglia are the purinergic receptors P2X_7_ and P2Y_12_ because they are found on M1 and M2 microglial phenotypes, respectively [26]. These receptors represent targets that could elucidate the pro-inflammatory (M1 phenotype) and anti-inflammatory (M2 phenotype) roles of microglia in neuroinflammation. Several PET radiotracers that target the P2X_7_ receptor have been investigated in preclinical studies [27,28,29,30,31,32], and recent efforts have also advanced P2Y_12_ receptor-targeted PET radiotracers to preclinical evaluations [26,33,34,35]. A promising neuroinflammation target is the glucose transporter (GLUT) 5, a high-affinity fructose-specific facilitative hexose transporter which represents the principal fructose transporter in the body [36]. In the brain, GLUT5 is predominantly expressed on microglia [37,38,39], and cerebral fructose metabolism has been identified as a potential driving mechanism in AD pathology [40]. Thus, GLUT5 represents a novel biomarker for PET imaging of neuroinflammation in neurodegenerative diseases [41]. A fluorine-18 labeled fructose derivative, 6-deoxy-6-fluoro-D-fructose (6-[^18^F]FDF), was developed for PET imaging of fructose metabolism in breast cancer via GLUT5 [42]. PET imaging studies with 6-[^18^F]FDF in breast cancer models also demonstrated the involvement of GLUT2, a low affinity transporter, in the uptake of 6-[^18^F]FDF [43,44], but the relative abundance of GLUT2 versus GLUT5 in brain is unknown. The present study seeks to determine if 6-[^18^F]FDF can be used to specifically image microglia in rodent models of neuroinflammation.

The most common radiotracer for PET imaging is 2-[^18^F]fluoro-2-deoxy-D-glucose ([^18^F]FDG) which is also a hexose. [^18^F]FDG-PET imaging generally captures inflammation as well as changes and differences in glucose metabolism in the brain, and is frequently used for imaging AD and related dementias for neuronal loss and neuroinflammation [45,46,47]. In this study, we compare PET imaging using 6-[^18^F]FDF with that using [^18^F]FDG in lipopolysaccharide (LPS) rat models of neuroinflammation in the context of our laboratory’s previously published results of imaging in this model with the 2nd generation TSPO PET radiopharmaceutical, *N*-acetyl-N-(2-[^18^F]fluoroethoxybenzyl)-2-phenoxy-5-pyridinamine ([^18^F]FEPPA) [48].

## 2. Results

### 2.1. Early Increase in 6-[^18^F]FDF Uptake in LPS-Injected Striatum

**Figure 1:**
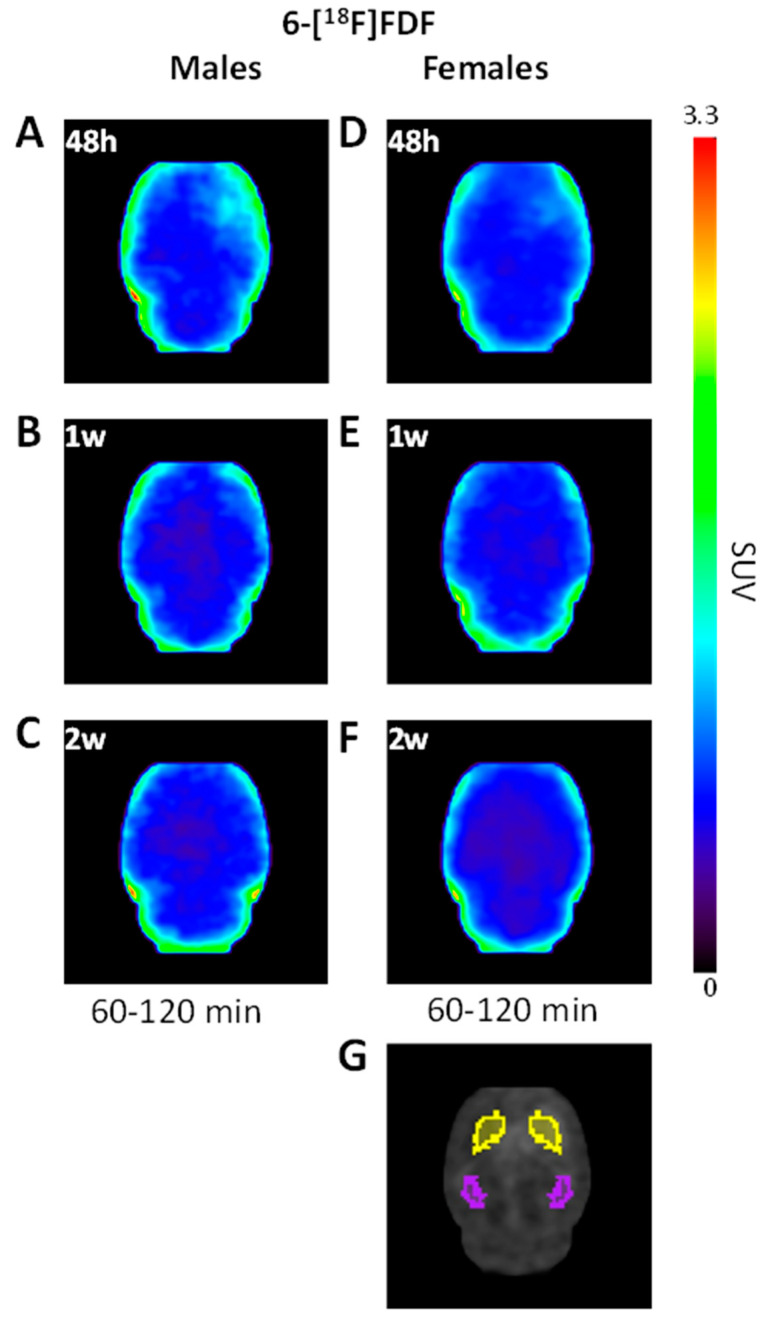
Representative static iterative transverse PET images of 6-[^18^F]FDF binding in unilateral lipopolysaccharide (LPS)–injected rat striatum in male (**A**–**C**) and female rats (**D**–**F**) at 48 h, 1 week, and 2 weeks post–LPS, and (**G**) MRI indicating the regions of interest of striatum (yellow) and hippocampus (purple).

**Figure 2:**
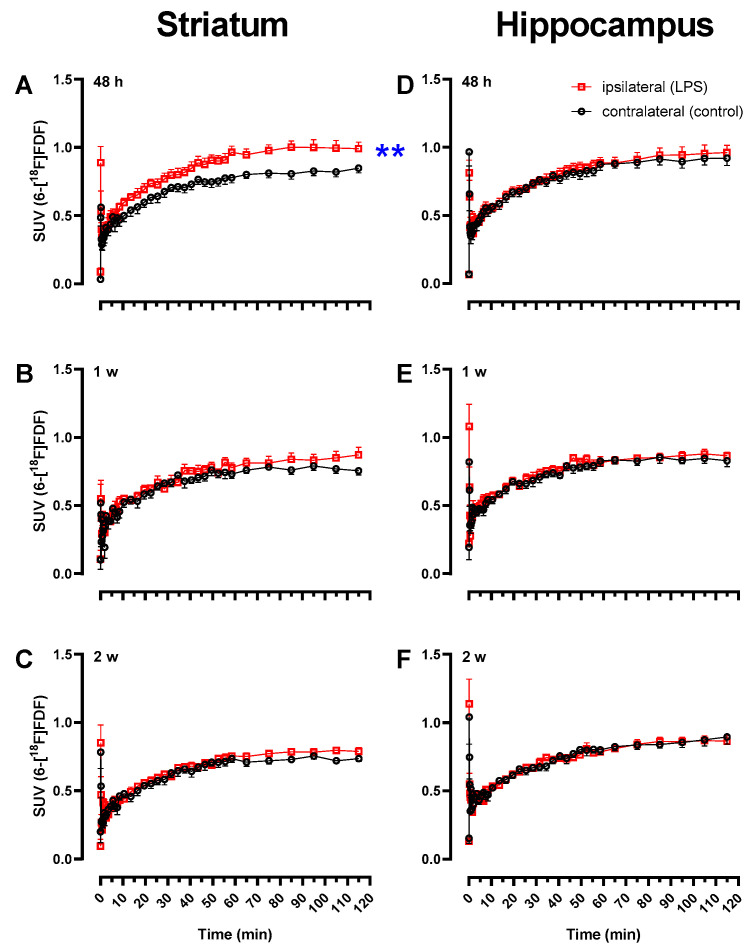
Time–activity curves (TACs) of 6-[^18^F]FDF in striatum and hippocampus of rats injected unilaterally with lipopolysaccharide (LPS) in the right striatum. Average TACs (±SEM) of 6-[^18^F]FDF are shown in combined male and female rats in the ipsilateral and contralateral side of striatum (**A**–**C**) and hippocampus (**D**–**F**) at 48 h (n = 4M/3F), 1 week (n = 2M/3F), or 2 weeks (n = 2M/3F), post-LPS injection. ** *p* = 0.003, right vs. left striatum at 48 h (repeated measures ANOVA).

Following injection in rats, 6-[^18^F]FDF accumulated slowly in brain parenchyma after the initial vasculature signal. Due to radiodefluorination of 6-[^18^F]FDF [42,49], bone accumulation of the radioactivity (see Figure S1 for an example) also increased with time, resulting in significant spillover of radioactivity from the skull to adjacent cerebral and cerebellar cortices, with time–activity curves (TACs) in the cortical areas showing increased radioactivity uptake throughout the 120 min acquisition similar to that in the skull. Nevertheless, in subcortical areas such as striatum, thalamus and hippocampus, the TACs plateaued 60 min after the bolus injection and remained stable at 0.8–1.0 standardized uptake values (SUV) for the remainder of the acquisition (see Figure 1 and Figure 2). In this proof-of-concept study, we focused on the striatum and hippocampus which had minimal skull spillover of radioactivity.

As shown in Figure 1, increased 6-[^18^F]FDF uptake was observed in the LPS-injected right striatum vs. the left side at 48 h post-surgery in both male (Figure 1A) and female (Figure 1D) rats. At later time points of one week (Figure 1B,E) and two weeks (Figure 1C,F), increased 6-[^18^F]FDF binding in the ipsilateral vs. contralateral striatum was largely diminished. Therefore, the early increase in 6-[^18^F]FDF uptake following LPS was in sharp contrast to that reported previously for the TSPO ligand [^18^F]FEPPA, which peaked at approximately one week post-LPS injection, and to that of the MAO-B ligand [^11^C]L-deprenyl, which developed after two weeks of LPS injection [48].

The results shown in Figure 1 were supported by TAC analyses depicted in Figure 2. Increased 6-[^18^F]FDF uptake (SUV, 60–120 min) in the ipsilateral striatum vs. contralateral side was observed at 48 h after surgery (0.985 ± 0.047 vs. 0.819 ± 0.033, n = 4M/3F, *p* = 0.0023; Figure 2A). Repeated measures ANOVA across the time course revealed a significant effect of LPS treatment (i.e., right vs. left striatum; *F*_1,6_ = 22.9, *p* = 0.003), time (*F*_38,228_ = 55.7, *p* < 0.0001) and time x brain region interaction (*F*_38,228_ = 2.81, *p* < 0.0001), suggesting significantly increased 6-[^18^F]FDF retention with time induced by LPS. No significant difference in TACs between left and right striatum was observed (n = 2M/3F) at one week (treatment: *F*_1,4_ = 6.17, *p* = 0.07 or time x treatment interaction: *F*_38,152_ = 0.96, *p* = 0.55; Figure 2B) or two weeks (treatment: *F*_1,4_ = 1.20, *p* = 0.34 or time x treatment interaction: *F*_38,152_ = 1.07, *p* = 0.38; Figure 2C) after LPS. As a control brain area, hippocampus did not show any significant left and right side difference in TACs at any time point studied (*p* > 0.05; Figure 2D,F), indicating the specificity of LPS-induced local response (see also Figures S2 and S3 for separate TACs of male and female rats).

**Figure 3:**
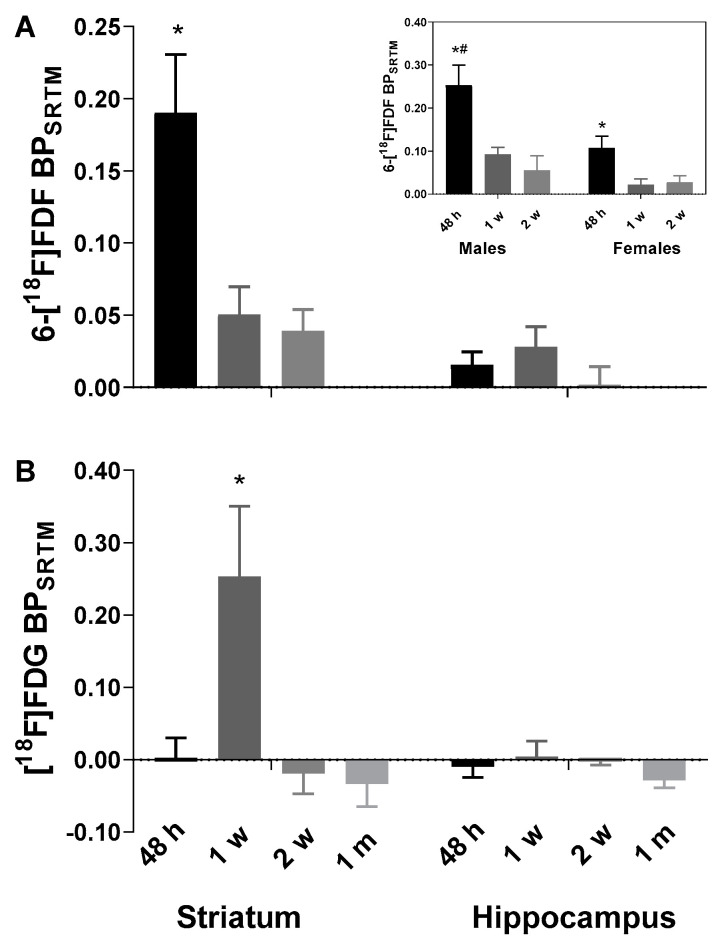
Binding potential (*BP*_SRTM_, ±SEM) of 6-[^18^F]FDF (**A**) and [^18^F]FDG (**B**) in unilateral lipopolysaccharide-injected right striatum of rats. *BP*_SRTM_ was derived from simplified reference tissue model (SRTM) using contralateral side as the reference tissue. Inset in (**A**) shows separate striatal 6-[^18^F]FDF data for male and female rats. * *p* < 0.05, 6-[^18^F]FDF at 48 h vs. other time points and [^18^F]FDG at one week vs. other time points in the striatum; ^#^ *p* < 0.05, male vs. female rats in 6-[^18^F]FDF uptake at 48 h (one-way ANOVA followed by Bonferroni corrections).

With the left-brain region as the reference, the binding potential (BP) on the right side was estimated with simplified reference tissue model (SRTM) [50]. As shown in Figure 3A, one-way ANOVA showed significant change of BP with time in LPS injected striatum (*F*_2,14_ = 7.68, *p* = 0.006), with significantly higher BP at 48 h (0.19 ± 0.11) compared to later time points. In preliminary analysis comparing male and female rats, a two-way ANOVA showed a significant effect of sex (*F*_1,11_ = 7.9, *p* = 0.017) and time (*F*_2,11_ = 10.3, *p* = 0.003) but not sex x time interaction (*F*_2,11_ = 1.57, *p* = 0.25), suggesting that male rats (0.25 ± 0.03; n = 4) had significantly higher BP in the ipsilateral striatum than the females (0.11 ± 0.03; n = 3) at 48 h after LPS injection but the response was later diminished with time in both sexes (Figure 3A, inset).

### 2.2. Increased [^18^F]FDG Uptake in LPS-Injected Striatum after One Week

**Figure 4:**
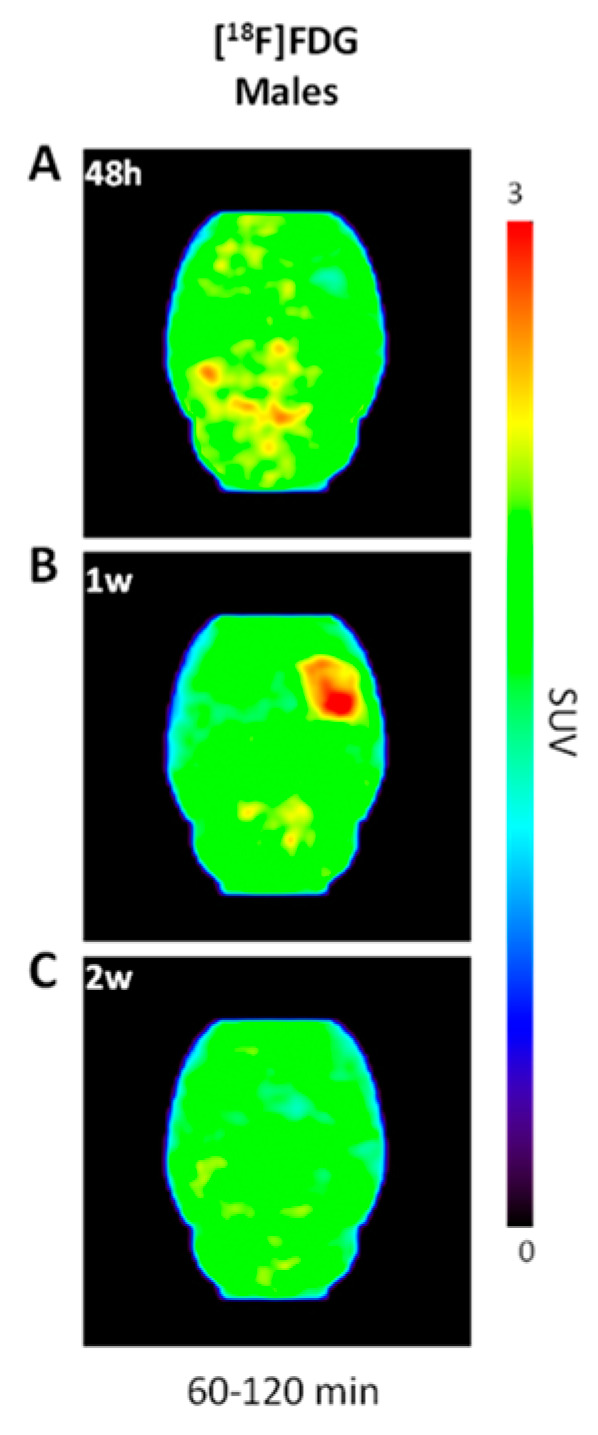
Representative static iterative transverse PET images of [^18^F]FDG binding in unilateral lipopolysaccharide (LPS)–injected rat striatum. Shown are PET images in male rats at (**A**) 48 h, (**B**) 1 week, and (**C**) 2 weeks post–LPS injection.

**Figure 5:**
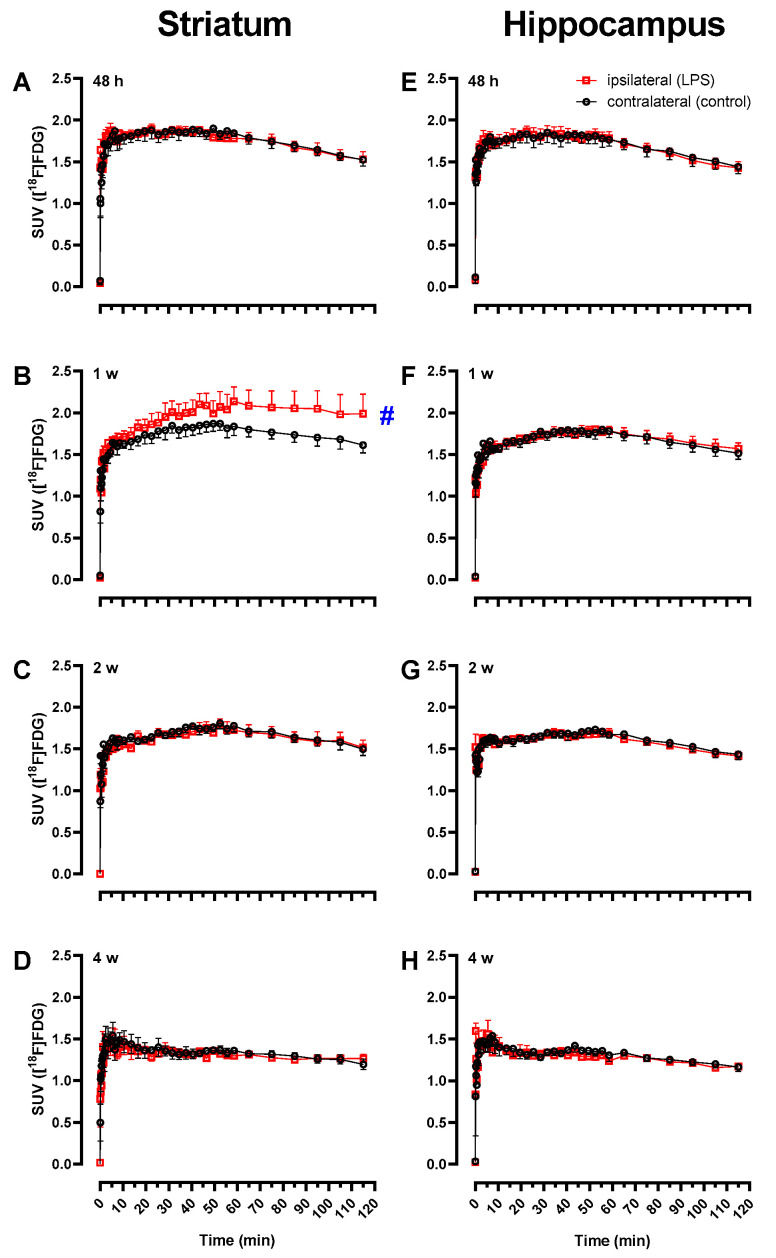
Time–activity curves (TACs) of [^18^F]FDG in striatum and hippocampus of male rats injected unilaterally with lipopolysaccharide (LPS) in the right striatum. Average TACs (±SEM) of [^18^F]FDG in ipsilateral and contralateral side of striatum (**A**–**D**) and hippocampus (**E**–**H**) at 48 h (*n* = 4), 1 week (*n* = 4), 2 weeks (*n* = 4), or 4 weeks (*n* = 3) post-LPS injection. ^#^ *p* = 0.05, right vs. left striatum at 1 week (repeated measures ANOVA).

We also performed dynamic PET imaging of [^18^F]FDG in the LPS rat model of neuroinflammation for comparison. As expected, [^18^F]FDG was rapidly taken up and retained in the rodent brain throughout the 120 min acquisition. PET scans in male rats following LPS-injection showed increased radioactivity accumulation at one week post-LPS (Figure 4B vs. 4A and 4C). At one week (Figure 5B), but not at other time points (Figure 5A,C,D), TAC analyses demonstrated a significant difference in the right vs. left striatum (*n* = 4; *F*_1,3_ = 10.0, *p* = 0.05) and time x brain region interaction (*F*_38,114_ = 1.58, *p* = 0.035), which is consistent with increased [^18^F]FDG retention local to the LPS-injected striatum. The control brain region hippocampus did not show any significant difference in the right vs. left striatum as indicated by the TACs at any time point (*p* > 0.05; Figure 5E–H). Accordingly, the BP for [^18^F]FDG in the ipsilateral striatum vs. contralateral side (Figure 3B) peaked at one week post-LPS injection (0.25 ± 0.06; *n* = 4), which was significantly higher than at other time points (one-way ANOVA *F*_3,11_ = 5.67, *p* = 0.014). This pattern of BP following LPS-injection into the right striatum is drastically different from that observed following 6-[^18^F]FDF injection, indicating that fructose and glucose metabolism are not occurring in the same population of cells.

### 2.3. Immunohistochemistry

**Figure 6:**
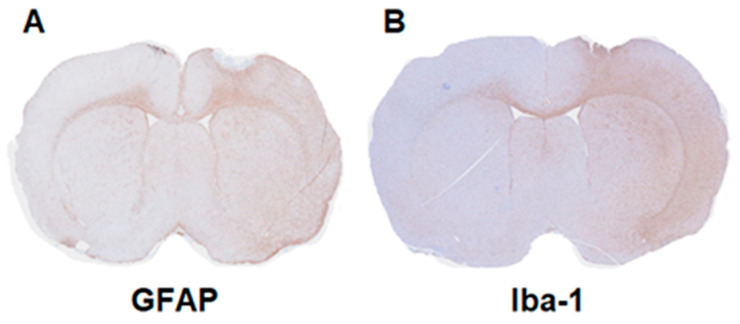
Immunohistochemical staining for the presence of microglia and astrocytes in unilateral lipopolysaccharide (LPS)-injected male rat brains. (**A**) Glial fibrillary acidic protein (GFAP, activated astrocytes) and (**B**) ionized calcium binding adaptor molecule 1 (Iba-1, microglia/macrophages) of an LPS-injected male rat brain at 7 days post-LPS injection in the right striatum.

Immunohistochemistry shown in Figure 6 demonstrates that an immune response was induced in our neuroinflammation model with activated microglia/macrophages and astrocytes being present on the ipsilateral side one week after LPS injection.

## 3. Discussion

The most common PET biomarker of neuroinflammation is TSPO; however, this target is not exclusive to microglia cells and many TSPO-targeted radiopharmaceuticals for clinical research imaging have been confounded by genetic polymorphisms [51], which complicates the interpretation of TSPO imaging in human brain. Novel PET radiotracers with the ability to specifically image activated microglial cells at different stages are needed to improve our understanding of the role of microglia in neuroinflammation and neurodegenerative diseases [10,11,12,13,14]. In the brain, GLUT5 is predominantly present on microglia [37,39,52], and represents, to our knowledge, an unexplored PET imaging biomarker of neuroinflammation. In this proof-of-concept study, we evaluated the suitability of 6-[^18^F]FDF, a substrate of microglia-located GLUT5, for PET imaging of neuroinflammation in rats injected with LPS into the right striatum and revealed increased radioactivity accumulation in the ipsilateral side compared to the contralateral side (Figure 1). Immunohistochemistry studies have shown that in widely used LPS-induced rodent models of neuroinflammation, microglia activation begins within hours then peaks within 1 to 2 weeks, depending on the biomarker selected for immunohistochemical staining, and then gradually dissipates [53,54,55,56]. Indeed, our longitudinal in vivo PET imaging studies found that radioactivity accumulation in the right striatum following 6-[^18^F]FDF administration was highest at 48 h and returned to baseline by 2 weeks post-LPS injection (Figure 2). Therefore, 6-[^18^F]FDF uptake peaks earlier and returns to baseline sooner than that of the TSPO tracer, [^18^F]FEPPA, in male LPS-injected rats [48]. This supports our hypothesis that 6-[^18^F]FDF is likely imaging an early stage of microglial activation, since microgliosis has been shown to start earlier than astrogliosis in response to LPS insults [57].

Our preliminary study showed a trend for sex differences in 6-[^18^F]FDF accumulation in the LPS-injected right striatum over time, with male rats having a greater response than the females. These preliminary results might indicate a difference in microglial response to neuroinflammatory insult with LPS between males and females. Our findings are supported by preclinical studies that have shown sex-related differences in microglial function, microglial expression levels during development, and microglial immune response to LPS injection [58,59,60,61]. PET imaging studies have also revealed sex differences in microglia. Another TSPO PET radiotracer, [^18^F]GE-180, revealed higher binding in female mice in a neurodegenerative mouse model for β-amyloid (AppNL-G-F) [62]. Consistently, in human studies, PET imaging with [^11^C]PBR28 showed higher TSPO binding in female healthy control subjects compared to males [63]. The mechanism underlying the sex difference is unknown but could be related to estrous cycle and hormonal status. Taken together, further studies are warranted to confirm the sex differences in male and female cohorts and to examine the effects of hormones on LPS-induced 6-[^18^F]FDF uptake.

[^18^F]FDG is the most widely used PET radiopharmaceutical and is employed for imaging glucose metabolism in neuroinflammation states, including in traumatic brain injuries [64], AD and related dementias [46,47]; however, [^18^F]FDG is not a specific radiotracer for studying inflammation since glucose metabolism of inflammatory microglial and astroglial cells is confounded by glucose metabolism in neurons and other cells in the brain tissue [65]. Interestingly, our studies show that [^18^F]FDG uptake at the site of LPS-injection peaked at 1 week in response to LPS insult (Figure 3B, Figure 5), which coincides with increased [^18^F]FEPPA binding [48]. We postulate that the early stage of neuroinflammation including proliferation of microglia and astrocytes is accompanied by increased energy demand thus glucose metabolism. However, [^18^F]FDG has high background uptake throughout the brain, and therefore subtle changes in glucose metabolism are not likely to be detected by PET imaging. Only male rats were employed in our [^18^F]FDG study as female rats are known to show variable uptake and metabolism with this radiotracer in the brain [66]. Given the sex difference in LPS-induced 6-[^18^F]FDF uptake, future work could consider if female rats respond differently than males to LPS challenge with [^18^F]FDG.

Limitations of this study include a relatively small number of animals examined and defluorination and/or known residual [^18^F]fluoride in the formulation of 6-[^18^F]FDF, as consistently reported in previous studies [49] (see also Section 4.1 below and Figure S4), which could reduce the suitability for translation for human brain PET studies in the present formulation due to proximity to the skull. However, despite bone uptake of [^18^F]fluoride in the skull potentially leading to spill over to adjacent brain regions (e.g., cortices), the partial volume effect was minimal in deep nuclei (e.g., striatum and thalamus) as judged by plateaued rather than continuously increasing TACs during the 120 min acquisition. Overall, 6-[^18^F]FDF was shown to be a promising PET radiotracer for specifically imaging fructose metabolism in microglia via GLUT5 in the brain, and has potential applications for PET imaging of neuroinflammatory and neurodegenerative diseases. We demonstrated the ability of 6-[^18^F]FDF to specifically image early microglial activation in a rodent model of neuroinflammation. Further studies could include more detailed histochemical examination of the expression of GLUT5 and other brain glucose/hexose transporters (e.g., GLUT2), including their cellular localization and relationship to other markers of glial activation (e.g., ionized calcium binding adaptor molecule 1 [Iba1] and glial fibrillary acidic protein [GFAP]) in models of neuroinflammation to correlate with 6-[^18^F]FDF imaging findings. Future studies with 6-[^18^F]FDF could examine PET imaging of neurodegenerative disorders that involve microglia including AD and Parkinson’s disease. Another area for future study includes drug addiction as GLUT5 expression and the density of resting microglia have been reported to be increased in brains of methamphetamine users [52]. Further studies could also examine whether differences in 6-[^18^F]FDF uptake among M1 and M2 microglial phenotypes exist. Pro-inflammatory M1 macrophages increase their glucose metabolism, while anti-inflammatory M2 phenotypes have significantly lower glucose consumption than M1 [67]. However, given the inherent heterogeneity of microglia under a pathological condition, in particular in vivo, the M1/M2 dichotomy of microglia might not capture the full picture of microglial status [68,69,70] and should be considered when developing new biomarkers for neuroinflammation.

## 4. Materials and Methods

### 4.1. Radiochemical Synthesis

Radiochemical synthesis of 6-[^18^F]FDF was performed as previously described [49]. Briefly, the methyl 1,3,4-tri-*O*-acetyl-6-*O*-(methylbenzene-sulfonyl)-α/β-*D*-fructofuranoside precursor (provided by Dr. Frank Wuest of University of Alberta, Edmonton, AB, Canada) was labeled using a [^18^F]KF/Kryptofix (K_222_) complex, followed by acid hydrolysis, and isolation by semi-preparative HPLC (Phenomenex LUNA C18(2) 10 μm 250 mm × 10 mm, 0.1 M sodium acetate buffer, pH = 5, at 2 mL/min). The collected peak was used directly for injection. Radiochemical identity was verified by HPLC and by co-spotting and staining by radio-TLC, and radiochemical purity was determined to be >90% (95:5 MeCN:H_2_O). The collected 6-[^18^F]FDF peak can be contaminated by a residual [^18^F]fluoride because of similar retention times on the established semi-preparative HPLC conditions (see Figure S4). [^18^F]FDG was purchased from Isologic Innovative Radiopharmaceuticals (ISOLOGIC, Toronto, Canada).

### 4.2. Lipopolysaccharide Rat Models of Neuroinflammation

Rat models of neuroinflammation were prepared by injecting LPS (L2630, serotype O111:B4; Sigma-Aldrich, St. Louis, MO, USA) unilaterally into the right striatum (caudate putamen) as previously described at our laboratory [48]. Briefly, adult male (6-[^18^F]FDF: 357–435 g, n = 4; [^18^F]FDG: 274–337 g, *n* = 4) or female (6-[^18^F]FDF: 207–219 g, n = 3) Sprague Dawley rats were anesthetized by isoflurane in O_2_ (5%, 2 L/min induction; 3%, 1 L/min maintenance) and positioned in a stereotactic head frame (David Kopf Instruments, Tujunga, CA, USA). Coordinates for the right striatum in relation to bregma were 0.5 mm anteroposterior, 3 mm lateral, and 5.5/4.5 dorsoventral [71]. The arm on stereotactic frame was maneuvered to the appropriate coordinates and a small hole was drilled at this location. A solution of LPS was injected at a rate of 0.5 µL/min via a microinjection pump with the microinjector placed at the appropriate coordinates for injection into the right striatum at a depth of 5.5 mm then 4.5 mm for a total of 50 µg in 4 µL injected.

### 4.3. Dynamic PET/MR and PET/CT Acquisition

PET/ magnetic resonance imaging (MR) or PET/ computed tomography (CT) was performed with 6-[^18^F]FDF in LPS rat models of neuroinflammation at 48 h (*n* = 4M/3F), 1 week (*n* = 2M/3F), and 2 weeks (*n* = 2M/3F) post-LPS injection, as well as with [^18^F]FDG in another cohort of male rats at 48 h (*n* = 4M), 1 week (*n* = 4M), 2 weeks (*n* = 4M) and 4 weeks (*n* = 3M) post-LPS injection. Two male rats in the 6-[^18^F]FDF study were sacrificed at one week post-LPS injection for immunohistochemistry and one male rat in the [^18^F]FDG study died after two weeks post-LPS. PET image acquisition following injection with the radiotracers was performed as previously described [48]. Rats were anesthetized by isoflurane in O_2_ (4%, 2 L/min induction; 1–2%, 1 L/min maintenance) for lateral tail-vein catheterization then transferred to a nanoScan™ PET/MR 3T or a PET/CT scanner (Mediso, Budapest, Hungary). Anesthesia was maintained throughout the imaging session while body temperature and respiration parameters were monitored closely. A scout MR or CT was acquired for PET field-of-view (FOV) positioning, then MR (gradient echo [GRE] multi-FOV and fast spin echo [FSE] 2D) or CT images were acquired for PET corrections of attenuation and scatter with the segmented material map and for PET/MR or PET/CT co-registration to define anatomic brain regions of interest. Rats were administered a bolus injection of 6-[^18^F]FDF (11.83–27.29 MBq) or [^18^F]FDG (15.39–23.15 MBq) through the tail-vein catheter and a 120 min scan was acquired.

### 4.4. PET Data Analysis

Acquired list mode data were sorted into thirty-nine three-dimensional (3D) (3 × 5 s, 3 × 15 s, 3 × 20 s, 7 × 60 s, 17 × 180 s, and 6 × 600 s), true sinograms (ring difference 84). The 3D sinograms were converted in 2D sinograms using Fourier rebinning [72] with corrections for detector geometry, efficiencies, attenuation, and scatter before image reconstruction using 2D filtered back-projection with a Hann filter at a cut-off of 0.50 cm^−1^. Static images of the complete emission acquisition (0–120 min) and in the time frame of 60–120 min were reconstructed with the manufacturer’s proprietary iterative 3D algorithm (6 subsets, 4 iterations). The static iterative images were used for PET and MR or CT co-registration (0–120 min images) and for presentation in figures (60–120 min images; Figure 1 and Figure 4). All data were corrected for dead time and were decay-corrected to the start of acquisition. Dynamic filtered back-projection images were used to extract regional brain TACs using a stereotactic MR atlas [73] following co-registration with subject’s MR (T2 weighted 2D FSE, TR 3971 ms, TE 87.5 ms) or CT image implemented in VivoQuant^®^ 2021 software (Invicro, Needham, MA, USA). SUV were calculated by normalizing regional radioactivity for injected radioactivity and body weight. Radiotracer BP in the right striatum was estimated with SRTM, using left striatum as the reference tissue, implemented in PMOD4.203 (PMOD Technologies, Zurich, Switzerland) [50]. TACs of the left and right hippocampus, which were not affected by LPS injection in the striatum, were analyzed as control.

### 4.5. Immunohistochemistry

Immunohistochemistry was performed to examine the expression of Iba-1 and GFAP in brains of LPS-injected male rats (*n* = 2) at 1 week post-LPS injection to confirm the presence of activated microglia and astrocytes, respectively, and also the successful injection of LPS in the right striatum. Brain tissue was fixed in 10% formalin for 48 h then embedded in paraffin and prepared in 4 µm sections onto microscope slides. Slides were dewaxed through changes of xylene, followed by hydration through decreasing grades of alcohol in water (100%, 95%, and 70%). Slides were blocked with 3% hydrogen peroxide, then antigen retrieval was performed with slides being heated at 98 °C in a microwave for 30 min for those being stained for Iba-1. Serum block was applied as directed by the MACH-4 Universal HRP-Polymer kit (Intermedico, BC-M4U534L), followed by incubation with a rabbit anti-Iba1 primary antibody or a rabbit anti-GFAP primary antibody (Abcam, Boston, USA) at room temperature for 1 h. Color was developed using DAB (Agilent Dako, K3468; Carpinteria, CA, USA) and counter stained with hematoxylin. Slides were dehydrated by reversing the rehydration procedure and sections were mounted with mounting medium (Leica, 3801120; Concord, ON, Canada). Slides were scanned with a slide scanner (Olympus, Slideview VS200; Tokyo, Japan).

### 4.6. Statistical Analysis

Data are represented as the mean ± SEM. Statistical analyses were performed by using StatSoft STATISTICA 7.1 (Tulsa, OK, USA). Differences in average SUV 60–120 min between left and right side were examined by paired Student’s *t*-test. Differences in 39-frame TACs between left and right side of the brain regions were examined by repeated measures ANOVA. Differences in BP across the time points following LPS injection and between sexes were examined by one-way or two-way ANOVA.

## 5. Conclusions

The major finding of this study is an increased response of 6-[^18^F]FDF to a local bacteria endotoxin insult in rat brain at 48 h post-surgery, suggesting that 6-[^18^F]FDF imaging of fructose metabolism via GLUT5 in microglial cells could be an early biomarker of neuroinflammatory reactions. The preliminary observation of sex differences in 6-[^18^F]FDF response in rats warrants further studies of hormonal influences on microglial reaction.

## Data Availability

The datasets during and/or analyzed during the current study will be made available from the corresponding authors on reasonable request.

## Supplementary Materials

The following are available online at https://www.mdpi.com/article/10.3390/molecules27238529/s1, Figure S1: Unstripped static 6-[18F]FDF image of Figure 1A (48 h post-LPS injection in a male rat), showing radioactivity accumulation in the skull; but also increased uptake in the LPS-injected right striatum vs. the left side. Figure S2: TACs of 6-[18F]FDF in striatum of male and female rats injected unilaterally with LPS in the right striatum. Average TACs (±SEM) of 6-[18F]FDF are shown in the right and left striatum of male (A–C) rats at 48 h (n = 4), 1 week (n = 2), and 2 weeks (n = 2), re-spectively, post-LPS injection and of female rats (D–F) at 48 h (n = 3), 1 week (n = 3), and 2 weeks (n = 3), respectively, post-LPS injection. * *p* < 0.05, right vs left striatum at 48 h (repeated measures ANOVA). Figure S3. TACs of 6-[18F]FDF in hippocampus of male and female rats in-jected unilaterally with LPS in the right striatum. Average TACs (±SEM) of 6-[18F]FDF are shown in the right and left hippocampus of male (AC) rats at 48 h (n = 4), 1 week (n = 2), and 2 weeks (n = 2), respectively, post-LPS injection and of female rats (D–F) at 48 h (n = 3), 1 week (n = 3), and 2 weeks (n = 3), respectively, post-LPS injection. Figure S4. Results of quality control of a 6-[18F]FDF production with residual [18F]fluoride. (A) Radio-HPLC chromatogram shows 6-[18F]FDF at 3.2 min and residual [18F]fluoride at 2.4 min; (B) Radio-TLC also shows 6-[18F]FDF (93.3%) at 78 mm and residual [18F]fluoride (6.7%) at 53 mm.

## Sample Availability

Samples of the compounds are not available from the authors.
