# Supplementary material for: PET Imaging of Fructose Metabolism in a Rodent Model of Neuroinflammation with 6-[18F]fluoro-6-deoxy-D-fructose
Source: Molecules. 2022 Dec 3;27(23):8529. doi: 10.3390/molecules27238529 (PMC9736258; doi:10.3390/molecules27238529)
Supplement: Supplementary file 1 [file molecules-27-08529-s001.zip › Supplementary Materials_6-FDF paper-Molecules-revised-new.pdf]

## Supplementary Materials

# PET imaging of fructose metabolism in a rodent model of neuroinflammation with 6-[$^{18}$F]fluoro-6-deoxy-D-fructose.

**Amanda J. Boyle[1,2*], Emily Murrell[1], Junchao Tong[1], Christin Schifani[1], Andrea Narvaez[1], Melinda Wuest[3], Frederick West[3,4], Frank Wuest[3,4], and Neil Vasdev[1,2*]**

[1] Azrieli Centre for Neuro-Radiochemistry, Brain Health Imaging Centre, Centre for Addiction and Mental Health, 250 College St., Toronto, M5T 1R8, ON., Canada

[2] Department of Psychiatry, University of Toronto, 27 King's College Circle, Toronto, M5T-1R8, ON., Canada

[3] Department of Chemistry, University of Alberta, Edmonton, T6G-2N4, Alberta, Canada

[4] Department of Oncology, University of Alberta, Edmonton, T6G-1Z2, Alberta, Canada

*Correspondence: amy.boyle@camh.ca, neil.vasdev@utoronto.ca Tel.: 416-535-8501 ext. 30884(A.J. B.); 416-535-8501 ext. 30988 (N.V.)

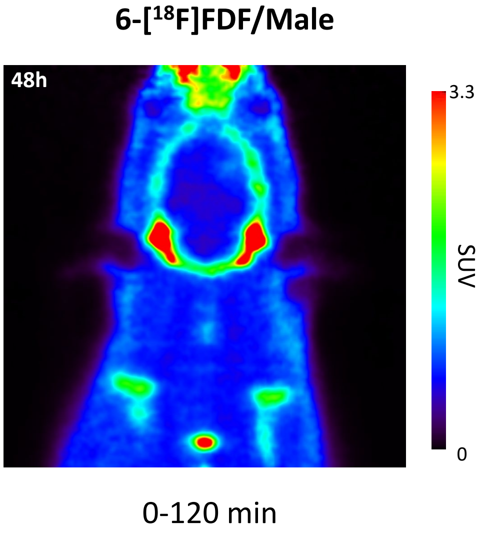


***Figure S1.*** Unstripped static 6-[$^{18}$F]FDF image of **Figure 1A** (48 h post-LPS injection in a male rat), showing radioactivity accumulation in the skull; but also increased uptake in the LPS-injected right striatum *vs.* the left side.

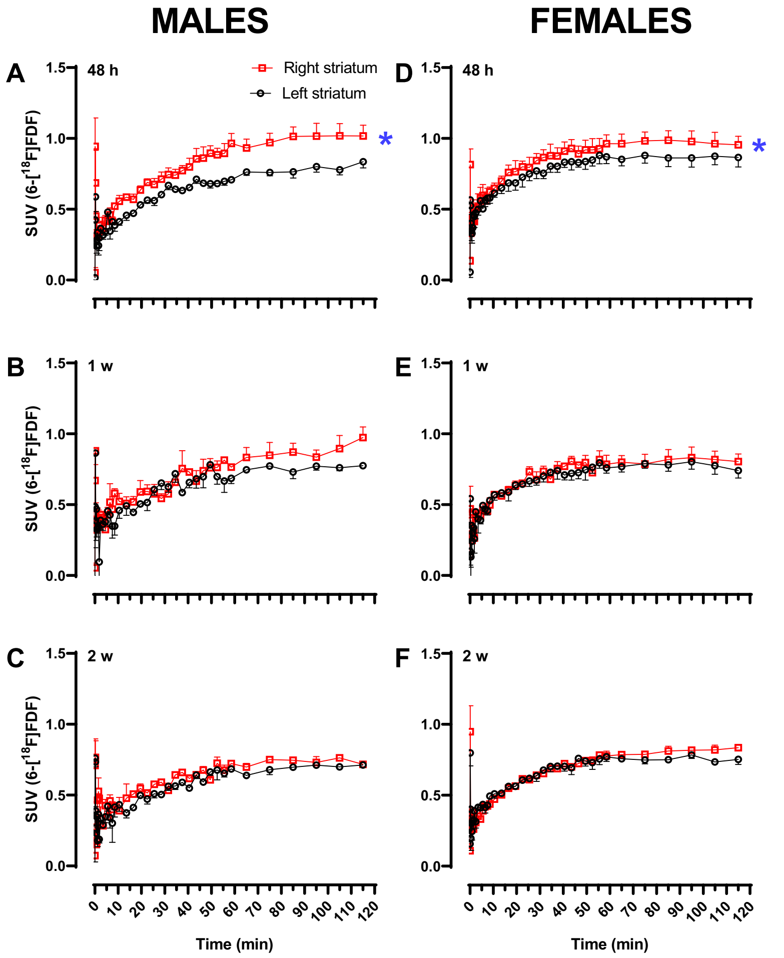


***Figure S2.*** TACs of 6-[$^{18}$F]FDF in striatum of male and female rats injected unilaterally with LPS in the right striatum. Average TACs (±SEM) of 6-[$^{18}$F]FDF are shown in the right and left striatum of male (**A-C**) rats at 48 hours (n=4), 1 week (n=2), and 2 weeks (n=2), respectively, post-LPS injection and of female rats (**D-F**) at 48 hours (n=3), 1 week (n=3), and 2 weeks (n=3), respectively, post-LPS injection. *$P$ < 0.05, right *vs* left striatum at 48 hours (repeated measures ANOVA).

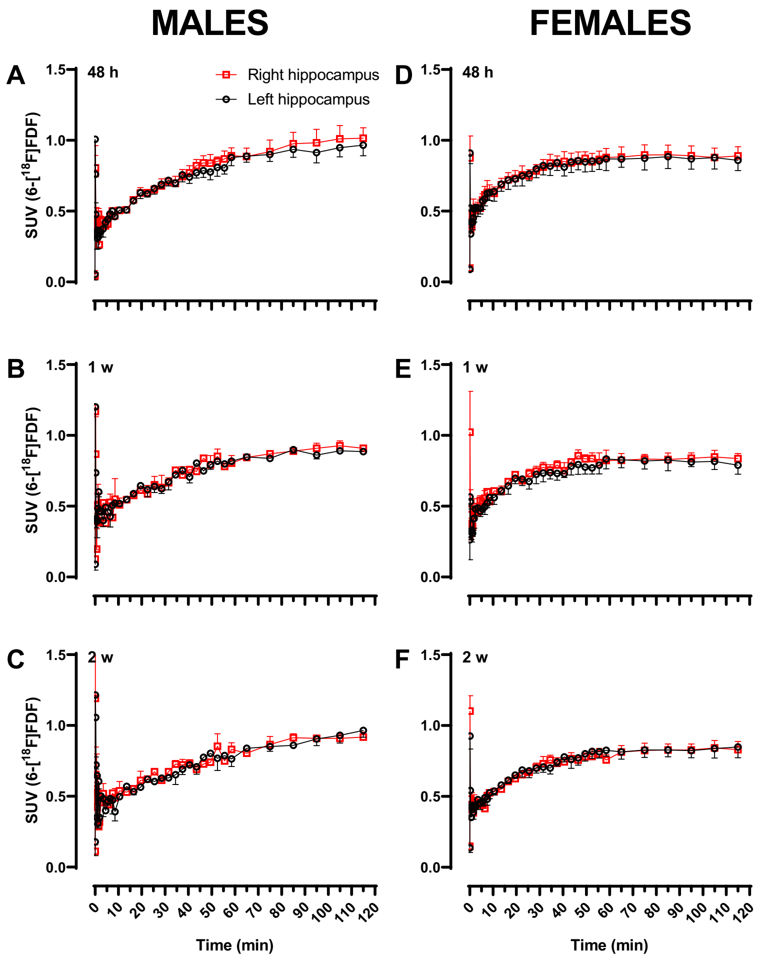


*Figure S3.* TACs of 6-[$^{18}$F]FDF in hippocampus of male and female rats injected unilaterally with LPS in the right striatum. Average TACs (±SEM) of 6-[$^{18}$F]FDF are shown in the right and left hippocampus of male (**A-C**) rats at 48 hours (n=4), 1 week (n=2), and 2 weeks (n=2), respectively, post-LPS injection and of female rats (**D-F**) at 48 hours (n=3), 1 week (n=3), and 2 weeks (n=3), respectively, post-LPS injection.

**A**

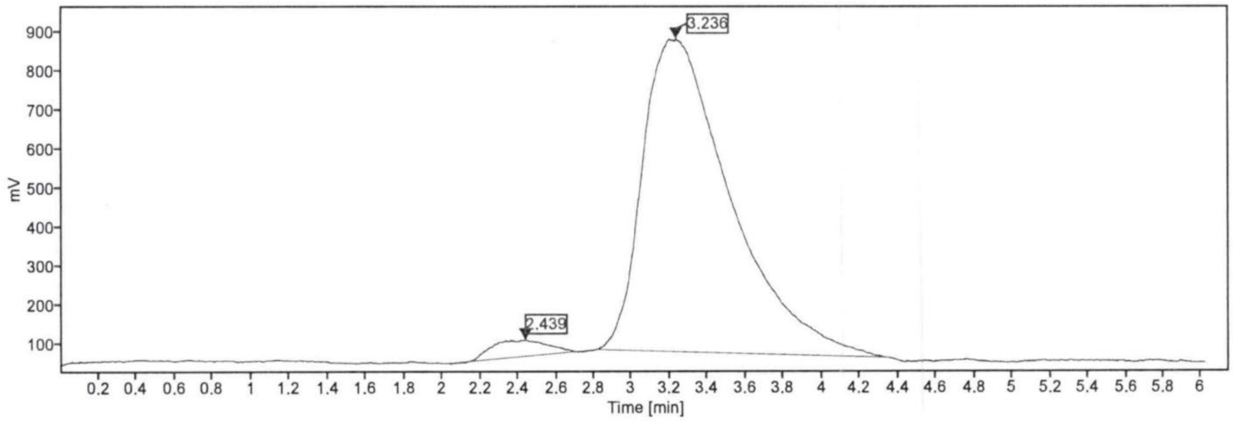


**B**

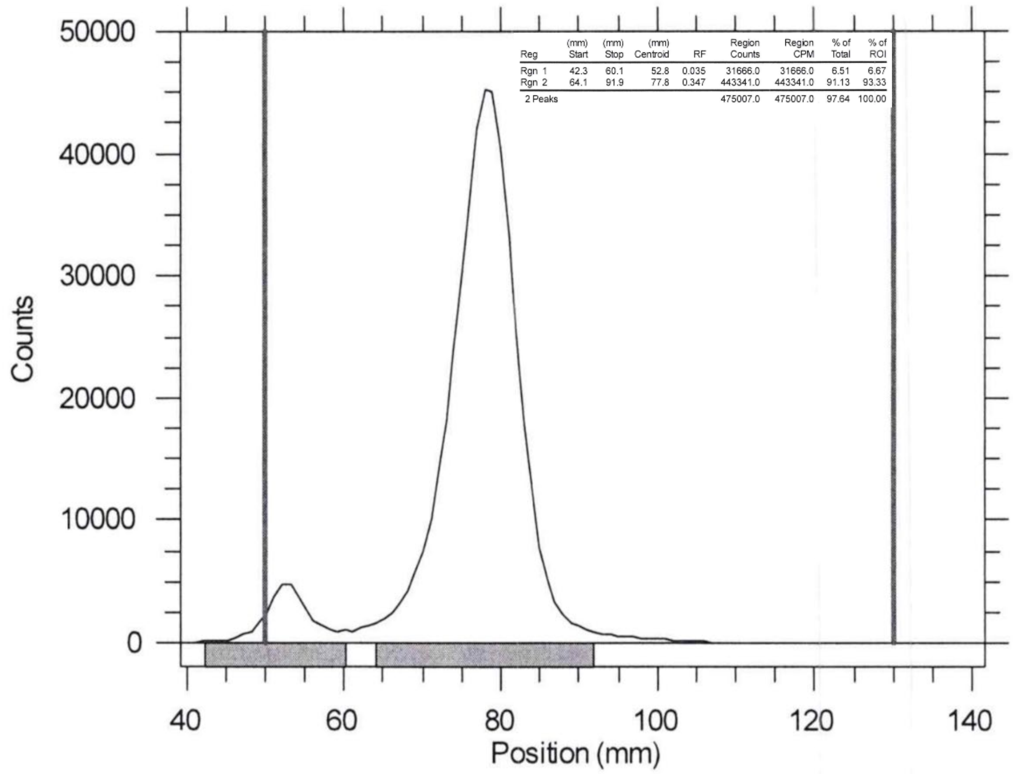


***Figure S4.*** Results of quality control of a 6-[$^{18}$F]FDF production with residual [$^{18}$F]fluoride. (**A**) Radio-HPLC chromatogram shows 6-[$^{18}$F]FDF at 3.2 min and residual [$^{18}$F]fluoride at 2.4 min; (**B**) Radio-TLC also shows 6-[$^{18}$F]FDF (93.3%) at 78 mm and residual [$^{18}$F]fluoride (6.7%) at 53 mm.
